# No Evidence of Robust Noun-Referent Associations in German-Learning 6- to 14-Month-Olds

**DOI:** 10.3389/fpsyg.2021.718742

**Published:** 2021-10-06

**Authors:** Jessica N. Steil, Claudia K. Friedrich, Ulrike Schild

**Affiliations:** Department of Psychology, Eberhard Karls University of Tuebingen, Tuebingen, Germany

**Keywords:** word comprehension, language acquisition, word learning, eye-tracking, infant cognition

## Abstract

Work with the looking-while-listening (LWL-) paradigm suggested that 6-month-old English-learning infants associated several labels for common nouns with pictures of their referents: While one distractor picture was present, infants systematically fixated the named target picture. However, recent work revealed constraints of infants' noun comprehension. The age at which these abilities can be obtained appears to relate to the infants' familiarity with the talker, the target language, and word frequency differences in target-distractor pairs. Here, we present further data to this newly established field of research. We tested 42 monolingual German-learning infants aged 6–14 months by means of the LWL-paradigm. Infants saw two pictures side-by-side on a screen, whilst an unfamiliar male talker named one of both. Overall, infants did not fixate the target picture more than the distractor picture. In line with previous results, infants' performance on the task was higher when target and distractor differed within their word frequency—as operationalized by the parental rating of word exposure. Together, our results add further evidence for constraints on early word learning. They point to cross-linguistic differences in early word learning and strengthen the view that infants might use extra-linguistic cues within the stimulus pairing, such as frequency imbalance, to disambiguate between two potential referents.

## Introduction

Where is the bottle? Researchers have been interested in the time when infants start to connect the articulated label “bottle” with its visual referent, for instance, a picture of the bottle. The investigation of such noun-referent associations has been of particular interest, as those seem to be a first step within the important milestone of word comprehension in infancy (Swingley, 2009; Johnson, 2016). The most upfront approach is to use a *parental report*, and simply ask infants' caregivers if they think their infant does or does not understand the word bottle. The MacArthur-Bates Communicative Developmental Inventory (MCDI; Fenson et al., 1994, 2007) is a widely adopted parental report on children's receptive and productive vocabulary. The accumulation of multiple MCDI reports across different age groups and languages provided a useful approach to get insight into infants' early word comprehension [see Wordbank Project (http://wordbank.stanford.edu; Frank et al., 2017)]. Overall, parents reported their infants understood first words around 8 months of life (Fenson et al., 1994). However, results from these subjective parental reports are not without challenges (for a full review please see Frank et al., 2021).

A promising tool to tackle early word comprehension more directly is to test the infant itself, for example with the *looking-while-listening paradigm* (LWL-paradigm; Fernald, 1985; Fernald et al., 2008; in other work referenced to as language-guided-looking: Bergelson and Swingley, 2012, or intermodal/crossmodal/infant preferential looking: Kartushina and Mayor, 2019). In the LWL-paradigm, infants see two visual stimuli (e.g., a picture of a bottle and a picture of a hat) presented side-by-side, whilst hearing an utterance that matches only to one of the two visual stimuli (e.g., “Look at the *bottle*!”). If an infant associates the word (“bottle”) with its referent (picture of the bottle), she is expected to look longer to the picture of the corresponding referent, compared to the distractor picture (Fernald et al., 2008; Swingley, 2012).

Mostly in the past decade, several studies used the LWL-paradigm to investigate English-learning infants within their first year of life. Infants listened, for example, to either “mommy” or “daddy” paired with videos of their own parents (Tincoff and Jusczyk, 1999), to either “hand” or “feet” paired with videos of unknown hands and feet (Tincoff and Jusczyk, 2012), or to common nouns like “banana” or “teddy” paired with prototypical pictures of respective referents (Bergelson and Swingley, 2012, 2015; Syrnyk and Meints, 2017). Even though all studies varied within their stimulus pairing and underlying analytic measures, the results suggest that the first signs of noun-referent associations in infants arise at 6–9 months in English-learning infants.

Yet, findings of the LWL-studies did not always neatly integrate with parental reports and were not always consistent across studies. They pinpointed word comprehension earlier in an infant's life than typically reported by parents (Fenson et al., 1994). Even more, several LWL-studies also showed a rather weak correlation between the individual performance of infants and respective parental report (Bergelson and Swingley, 2013, 2015, 2018; Syrnyk and Meints, 2017; Kartushina and Mayor, 2019, but see Styles and Plunkett, 2009). Several reasons can be accounted for the lack of accordance between evidence from parental reports and the LWL-paradigm, including subjective criteria for a word that parents count as understood (for a full review see Frank et al., 2021). However, not only parental reports of infants' early word comprehension could be biased, but the LWL-data also seem to be modulated by various aspects of the experimental setting. These should be considered before conclusions about early word comprehension abilities can be generalized.

So far, four experimental parameters have been identified to link to the variability of findings obtained with the LWL-paradigm, namely talker familiarity, target language, stimulus pairing, and infants' age. Within our study we did not systematically manipulate each of the following factors, however, they are all important for this study design and conclusions that might be drawn from the data.

First, the *familiarity of the talker* articulating the target words*:* Does it matter if the target words are articulated by a talker who is familiar to the infant (e.g., her parent) or by a talker who is unfamiliar to the infant? In terms of new word learning, a familiar talker (infant's mother) facilitated word learning in 24-month-old infants, while an unfamiliar talker did not (van Rooijen et al., 2019). This emphasizes, that maternal voice has a special role in early word learning. For word comprehension, the pattern of results is not entirely clear. It appeared that English-learning 6-month-olds' word comprehension was not impaired if an unfamiliar female talker produced highly socially relevant stimuli (Tincoff and Jusczyk, 1999, 2012). Furthermore, English-learning 8- to 9-month-olds showed word comprehension for common nouns produced by an unfamiliar female talker (Syrnyk and Meints, 2017). However, results from a recent study testing several age groups, suggested that an unfamiliar talker might hinder word comprehension (Bergelson and Swingley, 2018). Here, 8- to 10-month-old infants failed to associate the tested words with their referents when articulated by an unfamiliar talker, whilst 6- to 7-month-olds and 11- to 14-months-olds performed equally well, regardless of the familiarity of the talker. Infants' difficulties in word comprehension around 8–10 months were underpinned by an EEG-study. Here, 9-month-old Hungarian-learning infants understood nouns when articulated by a familiar talker, while they failed to do so when an unfamiliar talker articulated the nouns (Parise and Csibra, 2012). Together these results might imply a u-shaped development of understanding unknown talkers within the first year of life. In the present study, infants listened to an unfamiliar talker to further contribute data to this so far inconsistent picture.

Second, the *target language* might modulate infants' performance within the LWL-paradigm (Kartushina and Mayor, 2019). Up to now, most studies have exclusively investigated English-learning samples (Bergelson, 2020). Thus, little is known about potential cross-linguistic differences in the onset of word comprehension as evidenced by the LWL-paradigm. To our knowledge, only one study did target another population than English-learning infants (Kartushina and Mayor, 2019). In that study, Norwegian-learning infants aged 6- to 9-months did not associate nouns articulated by an unknown female talker with their corresponding referents until they were 8- to 9-months old, thus 2 months later than the previously investigated English-learning infants did (Bergelson and Swingley, 2018). This was especially striking as within the MCDI reports of parents of Norwegian-learning and English-learning infants did not report any remarkable differences in the number of words same-aged infants understood (see Kartushina and Mayor, 2019; Frank et al., 2021). As Kartushina and Mayor (2019) argue, the fact that Norwegian is phonologically more complex than English might have contributed to their findings. Importantly, these results highlight the need to assess word comprehension using LWL-studies within a broader range of target languages (Bergelson, 2020). Here, we investigate young infants with the target language German.

Third, the target words themselves and especially the *stimulus pairing* of these target words appear to modulate the infant's performance within the LWL-paradigm (Bergelson, 2020). Certain aspects of the stimulus pairing might increase or decrease infants' performance and might vary extensively across different LWL-studies (Delle Luche et al., 2015). Besides cues like perceptual similarity (Arias-Trejo and Plunkett, 2010) or semantic relatedness between target and distractor word (Bergelson and Aslin, 2017), word frequency of targets and distractors modulated infants' responses in the LWL-paradigm (Kartushina and Mayor, 2019). Here, Norwegian-learning infants succeeded only within stimulus pairs where one stimulus was from rather high word frequency, while the other was from rather low word frequency. To establish the word frequency experienced by children, the authors referred to the Child Language Data Exchange System (CHILDES; MacWhinney, 2000). The CHILDES database includes multiple transcripts of interactions between caregivers, experimenters and their children across a variety of target languages. Word frequency was estimated by counting how often a child heard a specific word. This absolute difference in word count for stimulus pairs (frequency imbalance) was positively related to the performance within the LWL-paradigm for 8- to 9-month-old Norwegian-learning infants listening to an unfamiliar talker (Kartushina and Mayor, 2019). Additionally, a comparable positive relation was found by Kartushina and Mayor (2019) in a *post-hoc* analysis of data from Bergelson and Swingley (2012) recorded from 6- to 7-month-old English-learning infants listening to their caregiver. Frequency imbalance could at least temporarily serve as an additional (extra-linguistic) cue, among semantic and contextual cues, that infants rely on during the LWL-task to succeed (Kartushina and Mayor, 2019). Before they have established robust word-referent mappings, infants might be more likely to map a frequently heard label to a frequently seen object, while they are more likely to map a less frequently heard label to a less frequently seen object. Within the present study, infants saw stimulus pairs with varying word frequency imbalance.

Lastly, we want to address *infants' age* and its relation to the performance within the LWL-paradigm. The previously introduced LWL-studies investigated infants ranging from 6 to 14 months of age (Bergelson and Swingley, 2012). At first sight, one would presume infants to show a gradual improvement in early word comprehension by age. This expected linear increase in word comprehension upholds for the parental reports. Not surprisingly, parents report infants to understand more words, as infants get older (Bergelson, 2020). Yet, such a gradual increase in performance is not consistently reflected within the LWL-paradigm. Not only did infants' age not always correlate to their performance within the LWL-paradigm (Bergelson and Swingley, 2012, 2015, 2018). Moreover, infants between 6- and 13-months showed little improvements within the LWL-paradigm and a more sizeable improvement not until 14-months of age (Bergelson and Swingley, 2012, 2015; Bergelson, 2020). This was the case at least for words articulated by a familiar talker. As already noted, for an unfamiliar talker the performance development might rather follow a u-shaped curve, which Bergelson and Swingley (2018) interpret in terms of a phonological restructuring of the lexicon (starting with less detailed representations, entries become more detailed over time, temporarily hindering the recognition of slight variation). Nevertheless, both studies suggest a non-linear development, which contrasts with the linear development that is reflected in the parental report (Bergelson, 2020). Here, we investigated infants across a broad age ranging from 6- to 14-months of age.

All in all, the foregoing research highlighted the importance of specifying early word learning for different target languages, age groups and experimental parameters, such as talker familiarity (Bergelson and Swingley, 2018) and word frequency differences within stimulus pairings (Kartushina and Mayor, 2019). With the present work, we planned to contribute data from 6- to 14-month-old German-learning infants listening to an unfamiliar talker. We directly tie in with the unfamiliar talker condition of Bergelson and Swingley (2018) and the study of Kartushina and Mayor (2019). Based on their previous findings, we expect to find word comprehension in measures of the LWL-paradigm within the tested age span. We test if German-learning infants show the onset of early word comprehension within the LWL-paradigm in parallel to English-learning infants, i.e., around 6- to 7-months of life (Bergelson and Swingley, 2018), or in parallel to Norwegian-learning infants, i.e., only later around 8- to 9-months of life (Kartushina and Mayor, 2019). Moreover, we address a potential u-shaped trajectory of word comprehension that English-learning infants showed given an unfamiliar talker (Bergelson and Swingley, 2018) within the German-learning sample and new stimulus material. Within exploratory analyses, we investigated the relationship between the word frequency imbalance of the tested stimulus pairs and infant's performance within the LWL-paradigm. If infants rely on additional frequency information for their success within the task, this should be reflected within a positive correlation between the frequency imbalance of the stimulus pairs and the performance in the LWL-paradigm (Kartushina and Mayor, 2019).

## Method

### Participants

The study was guided by the local ethics committee of the University of Tuebingen (11-14-2018, Friedrich_2018_1025_139). Data collection took place at the laboratory of the psychological institute in Tuebingen or at family's homes (~50%) between November 2018 and February 2020. All families were recruited in the area around the University of Tuebingen via e-mail, phone, and in person. Families participated in a larger study that investigated language development with multiple smaller experiments, one of them being the of interest LWL-paradigm. Families were compensated with a children's book (worth ~10 Euros).

In total, we tested 70 infants between 6 and 14 months of age^1^. We discarded data from 28 infants. Eleven of those infants heard another language than German at their homes and 17 infants contributed an insufficient amount of data (see Results). All 42 infants of the final sample (*M* = 10.24 months, *SD* = 2.63 months, 29 female) were raised monolingual, carried full-term (>37 weeks of pregnancy), and had no chronic ear infection at the time of testing. The highest maternal education level ranged from 10 educational years to a university degree. The majority (62%) of mothers completed a bachelor's degree or higher^2^.

To align the present analyses, we formed age groups based on Bergelson and Swingley (2018) with infants 6–7 months, 8–10 months, and 11–14-months. See Table 1 for further demographic information of the final sample distributed across the three age groups.

**Table 1:** Age in months (M, SD, range) and sample distribution in all four age groups.

	**Age group**		
	**6–7 months**	**8–10 months**	**11–14 months**
*N*_female_/*N*_total_	6/10	13/15	10/17
*M*_ageinmonths_	6.79	9.68	12.77
*SD*_ageinmonths_	0.63	0.89	1.49
Range_ageinmonths_	6.07–7.83	8.10–10.77	11.13–15.13^a^

a *Deviations are due to the procedure of calculating age in months with on average 30 days per month. However, all included infants fitted the age criterion at the time of testing*.

### Material

#### LWL-Paradigm

The LWL-paradigm (Fernald et al., 2008; Swingley, 2009) was implemented with Presentation^®^ (Neurobehavioral Systems Inc., Albany, CA, US) (Neurobehavioral Systems, 2020) and presented on an ASUS-Notebook (17.30 inch) with a screen resolution of 1,920 × 1,080 pixels. The Tobii X2-60 Compact^®^ (Tobii Technology AB, Stockholm, Sweden) collected infants' eye gaze with an average accuracy of 0.40 degrees under optimal conditions, sampling binocular from 60 Hertz (for further details see Tobii Technology AB, 2014).

We used 28 German disyllabic nouns with stress on the first syllable as target words (see Supplementary Materials, Table A1). Word selection was based on a parental screening questionnaire for 12-month-old infants (ELFRA-1; Grimm and Doil, 2006) supplemented by further words typically thought to be familiar to young infants, which had already been used in prior research (Bergelson and Aslin, 2017). All target words (audio length: *M* = 829.36 ms, *SD* = 25.54 ms) were embedded in carrier phrases (e.g., “Look at…”) and recorded by a male German native speaker (recording parameters: 16 bits, two channels, 44 kHz). The maximum mean audio intensity of our target words was 77.00 dB (*SD* = 2.19 dB, range = 71.90–81.20 dB). The talker was instructed to use infant-directed speech prosody. Infants received the auditory input over the laptop's speakers.

All 28 discrete pictures (see Supplementary Materials, Table A1), visualizing the target words, were acquired on Shutterstock.com (Shutterstock Inc., 2021). The LWL-task presented pictures of each stimulus pair simultaneously side-by-side (500 pixels left or right from the screen center) on a white background. The size of the target pictures was nearly constant (width resp. height = 700–800 pixels). We used videos of different objects (e.g., rattle) accompanied by different sounds (e.g., horn) as attention getters out of the Tobii Studio Software^®^ inventory. To center infants' eye gaze, a flashing dot was used (225 × 225 pixels).

#### Questionnaires

Within a *demographical questionnaire*, parents answered questions about their infant (age, sex, preemie, ear infection), about themselves (e.g., their highest school education, their native language, whether someone speaks another language than German with their infant), and (where applicable) about their partner (the same information as for themselves). Within the *vocabulary questionnaire*, the caregivers estimated their infant's receptive and productive vocabulary of 41 words, among them the 28 target words. Parents evaluated whether they believe their infant (i) “understands” or (ii) “understands and speaks” or (iii) “does not understand” the word. Additionally, parents rated their infant's daily exposure to each word. Parents were asked how often they believed their infant heard the word on a five-point Likert scale (“1” = “rarely” to “5” = “several times a day”). Mean values averaged over the whole sample on this five-point Likert scale were later used as the frequency measure for each target word.

#### CHILDES Sample

Kartushina and Mayor (2019) relied their word frequency measure on the absolute word count derived from the Norwegian CHILDES database (https://childes.talkbank.org/access/Scandinavian/; MacWhinney, 2000). To guarantee comparability we computed the frequencies of our targets based on the German CHILDES database (https://childes.talkbank.org/access/German/; MacWhinney, 2000). We included 64 transcripts of German-learning children (out of 119), as they contained between one and twenty-eight of our target words (*M* = 15.80 words, *SD* = 6.61) collected from a sample aged between 10.07 months and 12.17 years (*M* = 34.43 months, *SD* = 13.75 months). However, data for children from middle and late childhood appear to be a somewhat problematic measure of word frequency in the scope of the present study. For example, the absolute exposure to the word “diaper” might dramatically change for a 12-year-old child, compared to a 12-*month*-old child. Considering the whole Norwegian CHILDES database, children's age spanned only between 13.76 and 33.07 months (*M* = 24.93 months, *SD* = 4.64 months), leading to a more relatable age group for the tested infant sample. Therefore, we adjusted our CHILDES sample to a sub-sample of children under 33 months of age. This resulted in 51 children who heard between two to twenty-eight of our target words (*M* = 16.94 words, *SD* = 6.65 words) during their CHILDES observation. All children were between 10.07 and 32.95 months of age (*M* = 25.69 months, *SD* = 4.53 months). Given the provided project description (https://childes.talkbank.org/access/German/) individual observations ranged between 30 min and 4 h. Predominantly, they included one-on-one (semi-guided) play sessions between the primary caregiver and the child (e.g., Szagun, 2001). Only three children were reported to be observed during natural settings, such as waking up or breakfast situations (Wagner, 1985). These however were recorded almost 50 years ago.

### Procedure

The infant's caregiver gave their informed consent before the ~45–60 min long study started. In the following, we describe only the relevant aspects for the outlined research question. Parents completed the demographical and vocabulary questionnaire. For the LWL-task, the participating infant sat on the lap of her caregiver, who wore opaque glasses. Both faced a computer display (distance ~60 cm), either in the dimly lit lab or preferably in a dimly lit room at infants' homes. Before the LWL-task started, the two experimenters instructed the caregiver to interact as little as possible and not to talk to her infant during the task. Before starting the experiment, the experimenter started a five-point infant-adjusted eye-tracking calibration. By the calibration procedure, the geometric characteristics of the participants' eyes are estimated (as implemented in the Tobii Studio Software^®^). If necessary, the experimenter reran the calibration multiple times until the characteristics of the infant's eyes could be reliably extracted and used together with an internal, anatomical 3D eye model to calculate the gaze data (see www.tobiipro.com).

**Figure 1:**
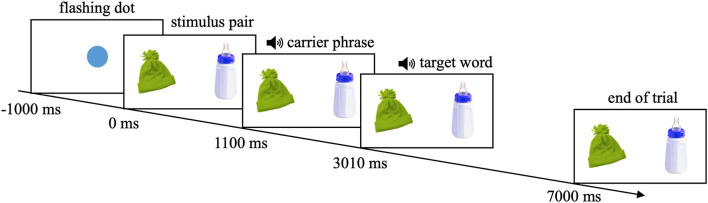
Time course of one trial (−1,000 to 7,000 ms) exemplified by the stimulus pair bottle-hat.

In total, infants saw 28 trials in the LWL-task. Each stimulus of every yoked stimulus pair was once named as the target. Thus, each stimulus pair was presented twice, once in each half of the experiment. Within one infant, both stimuli of a yoked stimulus pair remained on the same presentation side. Infants were randomly assigned to one of the four experimental orders, counterbalancing for the side on which the pictures occurred within each yoked stimulus pair (left vs. right screen side) and which stimulus was first named as the target within each yoked stimulus pair (Swingley, 2012). The sequence of the stimulus pairs within the experimental halves was randomized. The time course of one trial is illustrated in Figure 1 and was identical for the presentation of all stimulus pairs. All trials started automatically. The inter-trial interval was 1,000 milliseconds (ms). After every fifth trial, infants saw one of five different attention getters in randomized order, to keep their attention on the computer screen. In sum, the LWL-task took ~4 min and 30 s.

### Data Analysis

All analyses were conducted with the R software (R Core Team, 2021) and aligned with Bergelson and Swingley (2018). We conducted only non-parametric tests (two-sided Wilcoxon signed-rank test against chance, Kruskal-Wallis test), except for the Pearson correlation tests, and evaluated statistical significance based on the α-level of 0.05. We used the BayesFactor package to compute Bayes factors, with default priors of ttestBF, anovaBF, and correlationBF (Morey and Rouder, 2015). We reported BF_01_, hence the likelihood of the data given the null hypothesis (H_0_) relative to the alternative hypothesis (H_1_). Therefore, values greater one indicated evidence *for the null hypothesis* (H_0_). We used the evidence categories from Lee and Wagenmakers (2014), based on Jeffreys (1961), to interpret the strength of evidence. Example stimuli, pre-processed data, and R-scripts are available here https://osf.io/72tjz/?view_only=bb78b0c0a3e74b4b94c4dbd92c971317.

#### Eye-Tracking Data

For each sampling point (60 Hz) infants' looks were characterized as area of interest looks (left resp. right picture) or non-area of interest looks (e.g., off-screen). All looks over the post-target window of interest (368–3,505 ms after the onset of the spoken target word, due to technical restraints slightly modified [+/– 5 ms] but based on Bergelson and Swingley, 2018) were included in the following analysis. To control for extreme fussiness, we discarded all trials with infants looking <12.5% to the screen during each trial (per stimulus pair: *M* = 6.57 trials, *SD* = 3.34 trials). Due to insufficient data, we excluded 17 infants who provided data to <4 of our tested 14 stimulus pairs. This corresponds to less than one-quarter of all tested stimulus pairs, to keep data loss as minimal as possible.

To operationalize infants' looking preference, we calculated proportion indices (PI's). We expected infants who understood the target word within the LWL-task to look more at the target picture than at the distractor picture upon hearing the target word named (Bergelson and Swingley, 2012). The PI calculates infants' fixations to the target picture (relative to all fixations to the screen) in relation to infants' fixation to the same picture when it was a distractor (relative to all fixations to the screen; see Bergelson and Swingley, 2012). The PI could range from −1 to +1. More fixations to the target than the distractor picture within both presentations of the stimulus pair result in positive PI's and thus are interpreted as an indicator for noun-referent associations (Bergelson and Swingley, 2012). We reported only one PI per stimulus pair, because of the presentation of yoked stimulus pairs, the PI for each individual stimulus pair is arithmetically redundant (Bergelson and Swingley, 2012). In the following analyses, we operationalized noun comprehension by two different mean-measures: (i) proportion index by infant, calculated over all stimulus pairs (PI-by-infant), and (ii) proportion index by stimulus pair, calculated over all infants (PI-by-item).

#### Frequency Measures

For the parental frequency measure, we estimated the frequency imbalance within our stimulus pairs based on the parental report on word frequency within the vocabulary questionnaire. We computed the absolute frequency imbalance for each stimulus pair by subtracting the mean word exposure ratings of the 28 parental word evaluations within each stimulus pair. For the CHILDES frequency measure, we estimated the frequency imbalance, aligned to Kartushina and Mayor (2019). The absolute word count for each target word derived from the German CHILDES database via the childesr package (Braginsky et al., 2020).

#### Cluster Permutation

We computed a bootstrapped cluster-based permutation analysis using the eyetrackingR package (Dink and Ferguson, 2015). Similar to the cluster permutation analysis Kartushina and Mayor (2019) used, this analysis is based on the analysis proposed in Maris and Oostenveld (2007). We tested whether infants fixated the target above or below chance over the time course of the whole trial (0–7,000 ms). Therefore, we first created a chance data set, where looks to the target in relation to all looks on the screen were fixed to a chance-level of 0.50. We then tested our data against the at chance data using *t*-tests. For the dependent variable, we transformed the looks to the target compared to all looks to the screen via the arcsin square function to align it with the test assumption. Then, we estimated the size of clusters over the threshold of 2.02. Finally, we ran 1,000 simulations to test the probability of observing a cluster of the same or bigger size by chance (see Kartushina and Mayor, 2019). Clusters obtaining a probability below 5% were considered as significant. Aligned to previous analyses, we followed this procedure for the whole sample and the three age groups separately.

## Results

**Figure 2:**
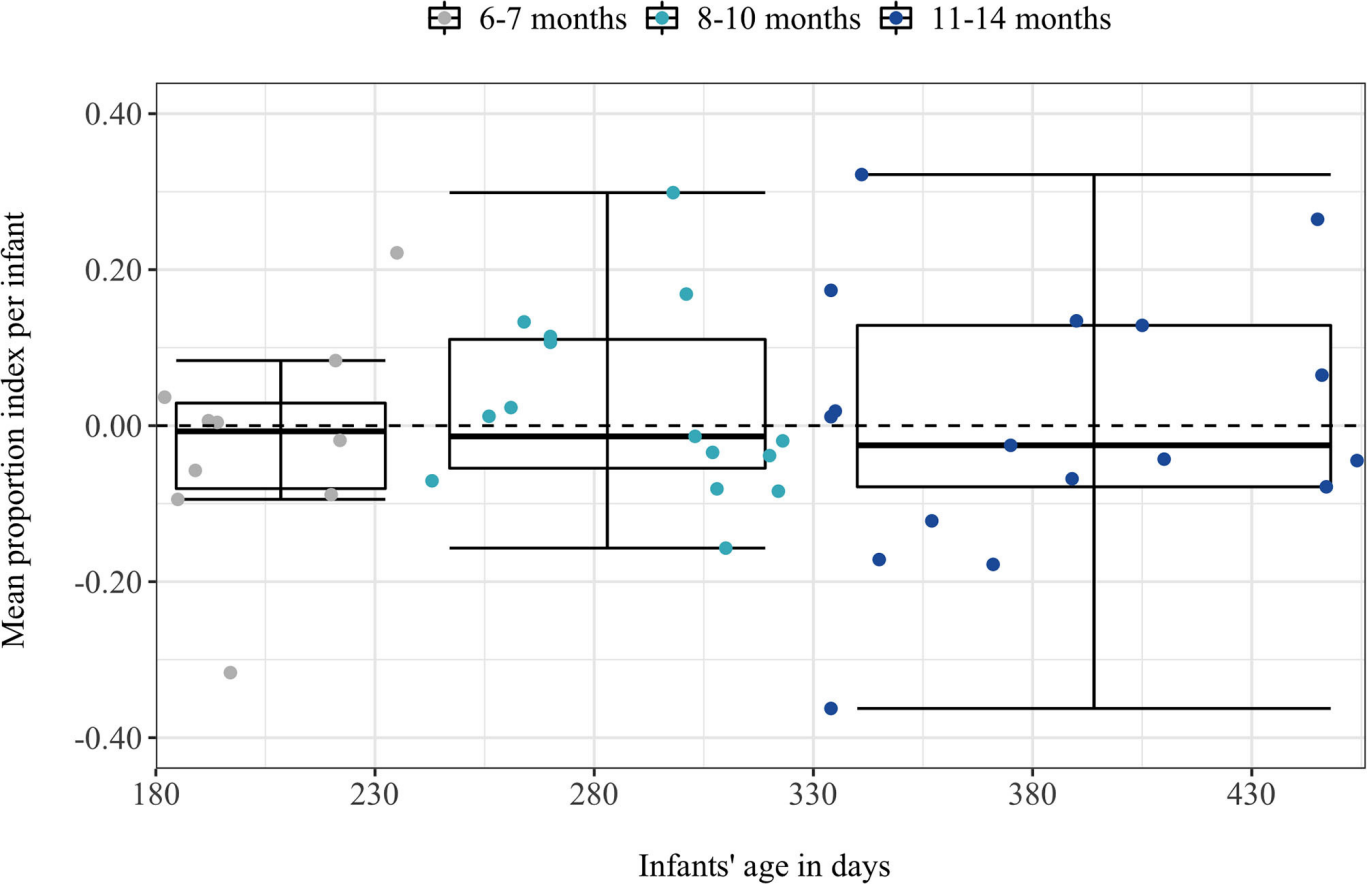
Mean proportion index per infant as a function of infants age in days and clustered into the three age groups. Age groups performance is indicated by the individual boxplots. Each dot corresponds to the individual performance of one infant.

**Figure 3:**
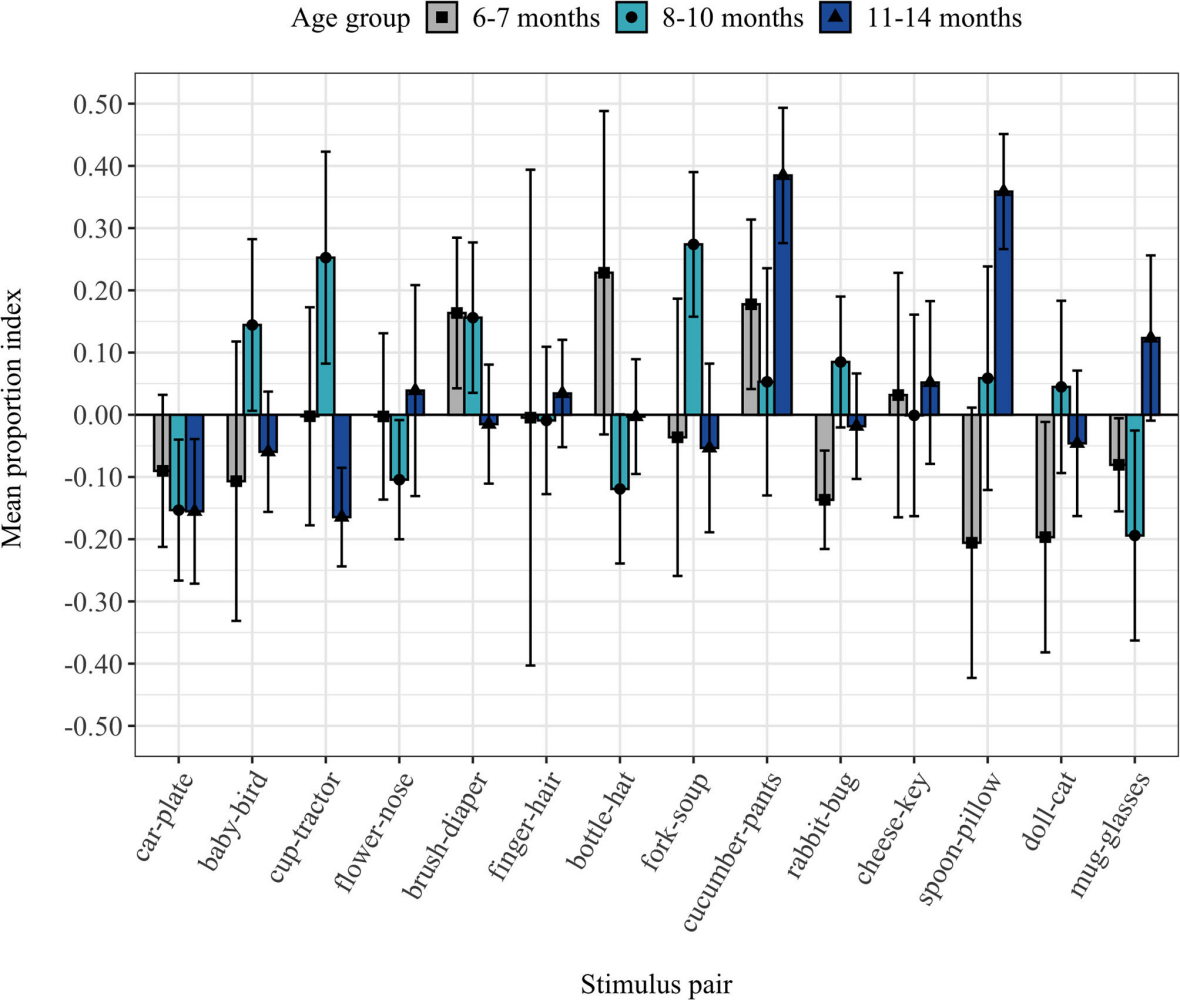
Mean proportion index by item (PI-by-item) for all age groups and standard errors.

Figure 2 illustrates the PI-by-infant measure. On average 7.67 stimulus pairs (*SD* = 2.77 stimulus pairs) were provided to calculate the PI-by-infant. In total 17 of 42 infants showed a positive PI-by-infant (*Mdn* = −0.016, *IQR* = 0.18). Contrary to our expectation, the mean PI-by-infant was not significantly different from zero, V = 442.00, 95% CI [−0.039, 0.046], *p* = 0.91, Cohen's *d* = 0.026, BF_01_ = 5.91 (moderate evidence for H_0_: PI = 0). Figure 3 illustrates the PI-by-item measure. On average 45% of infants (*SD* = 3.68 infants) provided data to compute the PI-by-item. In total, 7 out of 14 stimulus pairs showed a positive mean PI-by-item (*Mdn* = 0.0035, *IQR* = 0.046). Contrary to our expectation, the PI-by-item was not significantly different from zero, V = 59.00, 95% CI [−0.023, 0.074], *p* = 0.71, Cohen's *d* = 0.21, BF_01_ = 2.83 (anecdotal evidence for H_0_: PI = 0).

### PI-by-Infant and PI-by-Item Per Age Group

Unexpectedly, neither the PI-by-infant nor the PI-by-item differed significantly between the age groups, *PI-by-infant:* $\chi_{\left(2,N=42\right)}^{2}$ = 0.43, *p* = 0.81, BF_01_ = 4.66, *PI-by-item:* $\chi_{\left(2,N=42\right)}^{2}$ = 1.53, *p* = 0.47, BF_01_ = 3.83. In line with previous findings (Bergelson and Swingley, 2012), infants' age in days did not correlate with the PI-by-infant, *r*_(40)_ = 0.083, *p* = 0.60, BF_01_ = 2.57. Nonetheless, we conducted the planned separate statistical tests for each age group, to gain further insight into their noun comprehension. All descriptive and statistical results for each age group are displayed in Table 2. For none of the age groups, the PI-by-infant or PI-by-item differed significantly from zero, all *p*s ≥ 0.43. Furthermore, Bayes factors supported this null effect with anecdotal to moderate evidence, BF_01_ = 2.61–4.01.

**Table 2:** Descriptive distribution (Mdn) of both PI's, and two-sided Wilcoxon signed-rank test results, 95% CI's, Cohen's d, Bayes factor and evidence interpretation for all age groups.

		**Age group**		
		**6–7 months**	**8–10 months**	**11–14 months**
*N*_Positive_/*N*_Total_	PI-by-infant	5/10	7/15	8/17
	PI-by-item	4/14	8/14	6/14
*Mdn*	PI-by-infant	−0.0073	−0.014	−0.025
	PI-by-item	−0.020	0.049	−0.0090
Wilcoxon test	PI-by-infant	V = 22.00, *p* = 0.63	V = 67.00*, p* = 0.72	V = 74.00, *p* = 0.93
	PI-by-item	V = 39.00, *p* = 0.43	V = 65.00, *p* = 0.46	V = 53.00, *p* > 0.99
95% CI	PI-by-infant	[−0.12, 0.067]	[−0.047, 0.090]	[−0.083, 0.096]
	PI-by-item	[−0.10; 0.069]	[−0.054, 0.12]	[−0.056; 0.15]
Cohen's *d*	PI-by-infant	0.16	0.20	0.0086
	PI-by-item	0.14	0.24	0.21
BF_01_	PI-by-infant	2.90	2.93	4.01
	PI-by-item	3.29	2.61	2.84
Evidence for H_0_	PI-by-infant	Anecdotal	Anecdotal	Moderate
	PI-by-item	Moderate	Anecdotal	Anecdotal

### Vocabulary Questionnaire

Caregivers reported that their infants understood on average 11.55 words of the 28 target words (*SD* = 6.47 words, range = 0–24, Mode = 14 [5 out of 42 parents]). In line with previous studies (Bergelson and Swingley, 2013, 2015, 2018; Kartushina and Mayor, 2019), there was no significant correlation between the results from the LWL-task (PI-by-infant) and the number of target words reported as understood within the vocabulary questionnaire, *r*_(40)_ = 0.11, *p* = 0.50, BF_01_ = 2.36 (anecdotal evidence for H_0_: *r* = 0). The number of target words reported as understood increased with infants age in days, *r*_(40)_ = 0.46, *p* < 0.005, BF_01_ = 0.047 (moderate evidence for H_1_: *r* ≠ 0). This suggests a linear increase of infants' receptive vocabulary based on parental reports, which supports previous claims (Bergelson, 2020).

### Explorative Analysis: Frequency Imbalance

#### Word Frequency by Parent Report

**Figure 4:**
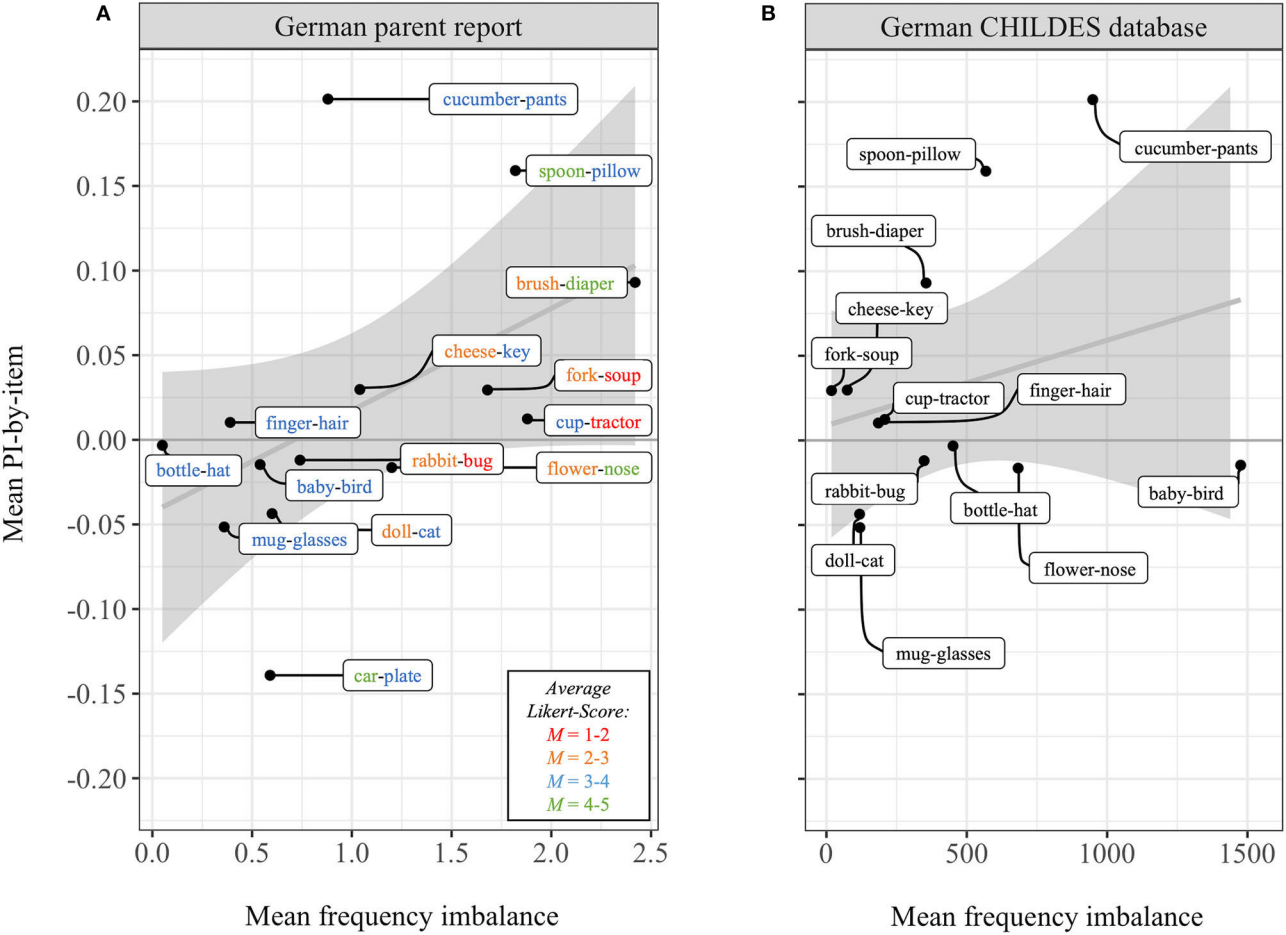
Mean frequency imbalance, based on **(A)** German parent reports of word exposure, and **(B)** German CHILDES database in relation to the PI-by-item for the whole sample. A higher mean frequency imbalance reflects a greater difference in word exposure between the words within each stimulus pair. Please note that due to the high deviation the stimulus pair “car-plate” is not displayed for the German CHILDES database.

On average, stimulus pairs' word frequency differed by 1.01 points (*SD* = 0.69 points) on the five-point Likert-Scale. The largest frequency imbalance was found for the stimulus pair brush-diaper with an average of 2.42 Likert points difference. “Diaper” was rated more frequently (*M* = 4.68), than “brush” (*M* = 2.26). The smallest frequency imbalance was found for the stimulus pair bottle-hat, with an average of 0.05 Likert points difference. “Hat” was rated slightly more frequently (*M* = 3.72), than “bottle” (*M* = 3.67). For a full display of word frequency for all 28 target words please see Table A1 in the Supplementary Materials. For the whole sample, frequency imbalance correlated significantly positive with PI-by-item, *r*_(12)_ = 0.49, *p* < 0.05, BF_01_ = 0.24 (moderate evidence for H_1_: *r* > 0). This indicates that stimulus pairs with higher frequency imbalance were associated with a higher PI-by-item. The frequency imbalance distribution of the parental report in relation to PI-by-item is shown in Figure 4A. Furthermore, we investigated the relation between frequency imbalance of stimulus pairs separately within each age group. For the age groups 6–7 months and 11–14 months no significant correlation resulted, all *r*s_(12)_ ≤ 0.047, all *p*s ≥ 0.49, BF_01_ = 0.49. Age group 8–10 months showed a significant positive correlation, *r*_(12)_ = 0.65, *p* < 0.01, BF_01_ = 0.20 (moderate evidence for H_1_: *r* > 0). This suggests that the positive relation between frequency imbalance of stimulus pairs and LWL-performance within the whole sample is mostly driven by the performance of the 8- to 10- month-old infants.

#### Word Frequency Imbalance by CHILDES Database

Car-plate had the highest frequency imbalance (Δfrequency = 5,278) and fork-soup had the lowest frequency imbalance (Δfrequency = 18). Table A1 within the Supplementary Materials illustrates target-specific word frequency measures. Surprisingly, the relation between the CHILDES frequency data and our PI-by-item was negative, *r*_(12)_ = −0.43, *p* = 0.94, BF_01_ = 2.36. However, this negative correlation was mostly driven by the high deviation of the stimulus pair car-plate (Δfrequency = 5,278). This absolute frequency imbalance was more than three times higher than the second-highest frequency imbalance of the stimulus pair baby-bird (Δfrequency = 1,476). Thus, when excluding the stimulus pair car-plate, the correlation with the LWL-task performance for the whole sample was positive, *r*_(11)_ = 0.27, albeit not significant, *p* = 0.18, BF_01_ = 0.34 (H_1_: *r* > 0). This is illustrated in Figure 4B. Regarding age groups separately, no significant correlation was evident between LWL-task performance and frequency imbalance, all *r*s_(11)_ ≤ 0.26, all *p*s ≥ 0.19, BF_01_ = 0.35–0.52. Importantly, the German CHILDES frequency measure did not significantly correlate with the frequency measure derived from the parental report, *r*_(12)_ = −0.21, *p* = 0.47, BF_01_ = 1.52.

### Explorative Analysis: Cluster Permutation

For the *total sample*, we identified three clusters: from 400 to 450 ms (*t* = 2.22), from 6,750 to 6,800 ms (*t* = 2.19), and the largest from 4,750 to 5,200 ms (*t* = 22.95). However, the probability of observing a cluster of equal or bigger size was on average 56% (range = 7.30–83.60%) for all three cluster. This suggests that the 42 infants did not fixate the target systematically above chance over the time course of all trials.

For infants between 6- and 7-months, we identified one cluster from 3,150 to 3,450 ms (*t* = −22.09). This would imply that during this time infants fixate the target below chance, thus preferably looked at the distractor. However, the probability of observing a cluster of equal or bigger size was 6% and therefore just above the predefined threshold of 5%.

For infants between 8- and 10-months, we identified four cluster from 3,000 to 3,050 ms (*t* = 2.03), from 3,300 to 3,350 ms (*t* = 2.04), from 4,900 to 4,950 ms (*t* = 2.37), and the largest from 6,750 to 7,000 (*t* = 15.06). The probability of observing a cluster of equal or bigger size was on average 70% (range = 20.90–91.50%).

For infants between 11- and 14-months, we identified five clusters: from 400 to 450 ms (*t* = 3.86), from 3,500 to 3,650 ms (*t* = −6.76) from 4,750 to 4,800 ms (*t* = 2.12), from 4,950 to 5,000 ms (*t* = 2.19), from 5,050 to 5,300 ms (*t* = 12.40). Again, the probability to observe a cluster of equal or bigger size was on average 62% (range = 26.50–84.70%).

## Discussion

The present study aimed to replicate previous results indicating that infants within their first year of life link nouns articulated by an unknown talker with corresponding visual referents (Bergelson and Swingley, 2018 for English-learning infants; Kartushina and Mayor, 2019 for Norwegian-learning infants). Unexpectedly, we failed to replicate these findings with *German-learning* infants. Neither the whole sample nor the individual age groups (6–7 months, 8–10 months, and 11–14 months) showed reliably more fixations to the target, in relation to the distractor picture within the used LWL-paradigm. This was further supported by the cluster permutation analyses, revealing that even when we consider the dynamic looks over the whole trial, infants did not consistently fixate the target above or below chance.

Within exploratory analyses, we investigated the relationship between the frequency imbalance of the stimulus pairs and the infants' performance on the LWL-paradigm. Here, we could replicate the finding that infants' performance within the LWL-paradigm was higher for stimulus pairs with a higher word frequency imbalance than for pairs with more or less the same word frequencies (Kartushina and Mayor, 2019). In line with previous work (Kartushina and Mayor, 2019), this was most evident for infants between 8- and 10-months.

Taken together, results of the LWL-paradigm suggest that German-learning infants between 6 and 14-months did not successfully link the 28 tested common nouns articulated by an unfamiliar male talker to corresponding visual referents. This contrasts with the evidence from the parental reports recorded for this sample, suggesting that infants understood at least some of these tested nouns. In the following, we introduce differences within the stimulus material, and general aspects of the stimulus pairings compared to previous studies, that might have affected infants' performance within the present work. Subsequently, we will evaluate alternative accounts for the relation between frequency imbalance and LWL-performance and address the surprisingly low performance of the 11- to 14-months-old infants and their potential implications.

### Characteristics of the Stimulus Material

First, contrary to previous studies with English-learning infants, we *only tested disyllabic words* here (as German vocabulary largely consists of disyllabic words, see for example, Domahs et al., 2008). Previous work mainly used monosyllabic target words (e.g., eleven monosyllabic words like “car,” four disyllabic words like “bottle,” and one trisyllabic word “banana” used by Bergelson and Swingley, 2012, 2018). Therefore, we might have underestimated infants' word comprehension, due to comparably more difficult disyllabic target words compared to monosyllabic target words used in former studies. That said, our parental vocabulary questionnaire suggested otherwise. Considering the age span from 6- to 9-months, parents of this German-learning sample reported even more “understood” words (27% of the target words) than parents in the study from Bergelson and Swingley (13% of the target words, 2012; 2018). This latter result is proposing that our tested target words were appropriate for the investigated age span. Even though, we took our targets from parental reports of infants' vocabulary, for some of our target words (e.g., “Suppe” [engl. soup]) most parents of our sample reported that their infant did not understand these words. Thus, the mean measure of 27% understanding cannot rule out the possibility that individual target words were not appropriate for the tested age span and therefore partly contributed to the lack of robust noun-referent associations within this study.

Second, as our stimulus material included low and high frequent words, our target words were on average reported as medium frequent (*M* = 3.29 on a Likert-scale from 1 to 5). This is comparable to the average frequency of 2.54 reported by the parents (on the Likert-scale from 0 = “never” to 5 = “very frequently”) in the previous study (Kartushina and Mayor, 2019). Yet, to assure that rarely heard target words do not account for our reported null finding, we performed an additional analysis (see Appendix B in the Supplementary Material). Here we excluded for each infant individually all stimulus pairs where parents reported one or both targets as “rarely” heard and repeated our analysis. In total, the previously reported pattern upheld: There was no evidence for robust noun-referent associations within this German-learning sample.

Third, in contrast to the conceptual replication (Bergelson and Swingley, 2018; Kartushina and Mayor, 2019), we used a *male* unfamiliar talker instead of a female unfamiliar talker. Neighboring research fields propose that female and male voices might differently affect infants' responsiveness (Richoz et al., 2017; Sulpizio et al., 2018), thus making it likely that the gender of the talker might modulate infants' attention during the LWL-task. On the other hand, already 7.5-month-old infants were capable to form word representations that are independent of the speaker's gender (van Heugten and Johnson, 2012). This makes it rather unlikely, that the gender of the speaker alone contributes to the lack of noun comprehension in the investigated sample. As no previous LWL-study has addressed this before, it is uncertain if and in what manner the gender and possible associated voicing characteristics of the unfamiliar talker shaped the results within the present work.

### Aspects of Stimulus Pairing

First, the *semantic relation* of the target and distractor word can hinder infants' performance in the LWL-paradigm. In a previous study by Bergelson and Aslin (2017), 6-month-old English-learning infants failed to associate a target with its referent picture (for instance, the target “mouth” articulated by their parent) when the picture was paired with a picture of a semantically related distractor picture (for instance a nose). Yet, the same infants successfully fixated the target within a semantically unrelated condition, i.e., the target picture of a mouth paired with the picture of a ball. Contrary to previous studies using words from different word categories within the stimulus pairs (Bergelson and Swingley, 2012, 2018; Kartushina and Mayor, 2019), three of our used stimulus pairs (e.g., rabbit-bug, finger-hair, spoon-pillow) comprised two words out of the same word category (see http://wordbank.stanford.edu for word categories; Frank et al., 2017). Consequently, this might have impaired word comprehension within these stimulus pairs and further challenged the whole performance within the task (Bergelson and Aslin, 2017). However, mean performance within these same-category stimulus pairs (PI-by-item: *M* = 0.052, *SD* = 0.09) was not lower compared to the different-category stimulus pairs (PI-by-item: *M* = 0.009, *SD* = 0.09). Neither did it systematically co-occur with other aspects of stimulus pairing, such as a high-frequency imbalance of the stimulus pair estimated by the parental report (same-category frequency imbalance: *M* = 0.98, different-category frequency imbalance: *M* = 1.02).

Second, as reported, the explorative analysis showed increasing performance within the LWL-paradigm for stimulus pairs with increasing *frequency imbalance rated by the parents*. Bayes factors suggested moderate evidence for this relation. Moreover, if age groups were tested separately, this correlation upheld only for infants between 8- and 10-months. This is in line with the previously investigated Norwegian-learning sample (Kartushina and Mayor, 2019), and thereby fits the claim that not all age groups reliably use frequency imbalance as an extra-linguistic cue to recognize referents of spoken words (Kartushina and Mayor, 2019; Frank et al., 2021). However, our findings differ from the previous findings, as we replicated this relation only for the frequency measure derived from the parental report and not for the measure derived from the German CHILDES database (as Kartushina and Mayor, 2019 did for their findings and the Norwegian CHILDES database). Moreover, for the stimuli of our study, parental reports and German CHILDES data did not positively correlate. This might reflect the different operationalization that both measures use to assess word frequency. In the following, we want to address this lack of accordance and raise a potential explanation.

How frequently parents use a specific word within a communicative situation that enters a database might be highly influenced by external situational factors. Specific situations are not representing the entire vocabulary. Specific words tend to co-occur dominantly in specific rooms (Custode and Tamis-LeMonda, 2020) and during specific activities (Tamis-LeMonda et al., 2019). Thus, when documenting a guided play interaction in the experimenter's lab, it is unlikely to hear the word “diaper” frequently, even though it is frequently used in the everyday life of the infant. As the German CHILDES data (https://childes.talkbank.org/access/German/) do not cover day-long observations but only some specific situations, one might question their representativeness. Furthermore, personality or demographical factors of communicative partners such as the maternal education level (Hart and Risley, 1995) might influence which words enter a database, and these characteristics might not match those of the parents of the here tested sample. Given the limited possibility to control for such potentially influencing confounding factors, we assume that the operationalization of word frequency based on the parental report of the tested infants is the better option. Indeed, comparing the frequency imbalance estimates of the tested 42 parents to the frequency imbalance estimates of additional 105 parents of infants within the same age span (parents filled out the same vocabulary questionnaire, but infants did not participate in the LWL-study) revealed a high agreement between both parental groups, *r*_(12)_ = 0.96, *p* < 0.001.

To further strengthen the evidence for the relationship between frequency imbalance based on parental reports and the performance within the LWL-paradigm, we reanalyzed the data of Kartushina and Mayor (2019). Surprisingly, within their data, frequency imbalance based on parental report correlated significantly *negatively* to the performance within the matching LWL-paradigm trials of the 8- to 9-month-old infants, *r*_(6)_ = −0.73, *p* = 0.040. This is exactly the opposite of our observation and might be due to a non-linear relation between their parental reports and the CHILDES frequencies in the study with Norwegian-learning infants. In these former data, high frequent words tended to show a ceiling effect within the parental report. This might have been an effect of the specific parental report that was used. Parents judged word frequency on a six-point Likert scale by considering word exposure since birth (and not current daily word exposure like we asked for in the present study). The ceiling effect might have underestimated actual frequency differences for some trials, which could account for the obtained negative correlations between parental reports and Norwegian CHILDES data on the one hand, and with LWL data of the Norwegian-learning infants on the other hand. Even though we argue that for the scope of our study parental reports seem to be the more appropriate choice of operationalizing how frequently infants heard the target words, this cannot be generalized in the way of an overall superiority of this measure. Undoubtedly, there lies great potential in measuring word frequency by the actual use within day-long parental speech. Just asking parents how often they *think* they use a word like we did, is prone to aspects like parental believes, answer tendencies, or the rather subjective nature of our questionnaire. Further work is needed to clarify potential differences in the operationalization of word frequency and combined measures that converge to similar results should be aimed for.

### Alternative Accounts: Frequency Imbalance and Exclusion Strategy

As already discussed, we found no evidence of robust noun-referent associations in the tested sample of 6- to 14-month-old German-learning infants. Nevertheless, performance was enhanced for our frequency imbalanced stimulus pairs, in particular for infants between 8 and 10 months. This might reflect the already introduced assumption of a strategy according to which infants map often heard words to often seen objects (and vice versa). The frequency imbalance might help infants as a cue to detangle the target from the distractor stimuli without reflecting specific word-referent pairings in the infants' proto-lexicon (Kartushina and Mayor, 2019). One might speculate that frequency-based matching might be a strategy that infants temporarily use until they figure out more effective ways of matching words and referents. When they switch their strategy to one-on-one mapping of word and referent, very young children might temporarily show somewhat worse performance, because they have not stored enough word-referent associations. Thus, next to the phonological restructuring of the proto-lexicon (see Bergelson and Swingley, 2018), alternative explanations based on different learning strategies might also be considered for the interpretation of u-shaped word comprehension trajectories in infant LWL-data. Nevertheless, our study design and in particular the inconsistencies found for different frequency measures (see above) do not allow us to make strong conclusions about different strategies used across infancy and very young childhood. Furthermore, neither the present nor the study by Kartushina and Mayor (2019) measured the actual occurrence of referent objects. Object-copresence, however, is an important predictor for noun comprehension (Bergelson and Aslin, 2017). Just hearing a word repeatedly, without seeing its referent should not elicit the assumed enhanced effect on LWL-performance (Kartushina and Mayor, 2019). Within this notion, object-copresence is somehow presumed in our studies, without properly testing or controlling for such.

Alternatively, the relation between frequency imbalance of stimulus pairs and LWL-performance could also hint to an *exclusion strategy* that infants apply. The use of yoked picture pairs in the typical LWL-paradigm might enable an infant to succeed if she knows only one of the two noun-referent associations (Bergelson and Swingley, 2012). When an infant for instance sees the stimulus pair “bottle-hat,” while hearing “bottle,” she might already associate the word with the correct picture of the bottle. However, seeing the stimulus pair bottle-hat, while hearing the word “hat,” she might not know the correct referent. Based on the association between “bottle” and its referent, she can rule out the only other option and fixate the correct picture of the hat. Especially for stimulus pairs with a high frequency imbalance, it is likely that infants know only one of both labels (namely that of the high frequent target word) and rely on such an exclusion strategy. This said, the use of such an exclusion strategy should imply a slightly different correlation pattern than the one reported in this study. Relying on the exclusion strategy, infants' LWL-performance should be enhanced not just for frequency imbalanced picture pairs, but also for frequency balanced picture pairs with two rather high frequent stimuli. Within our study, there were no balanced stimulus pairs consisting of two high frequent target words. However, performance for most balanced stimulus pairs consisting of two medium frequent words showed at chance performance (see Figure 4A all pairs with blue font only). Following the exclusion strategy, we would expect a positive correlation between the highest frequent word within the stimulus pair and the LWL-performance. For our data, this correlation was weaker with *r*_(12)_ = 0.35, than the correlation between frequency imbalance and LWL-performance and failed to reach significance, *p* = 0.20, BF_01_ = 1.06.

Previous work on the mutual exclusivity strategy applied by very young children also somewhat challenges the interpretation of the present data in terms of an exclusion strategy. This strategy is assumed to discourage an infant in assigning a second label to a single object. In respective research, very young children learned a novel label for a novel object. Following the learning phase, they saw the newly learned object together with a novel object while they heard a novel label. Seventeen-month-old infants demonstrated a mutual exclusivity strategy. They fixated the novel object after listening to the novel label. By contrast, 14-month-olds did not apply an exclusivity strategy. They preferably looked at the familiar (newly learned) object, after listening to the novel label (Halberda, 2003). If the here reported positive relation between frequency imbalance and LWL-performance is indeed driven by infants exploiting their knowledge of high frequent words and drawing conclusions to the unknown object and its label, this would suggest that a precursor of this mutual exclusivity strategy might be available earlier than previously reported (Halberda, 2003; Bion et al., 2013). Alternatively, the mutual exclusivity strategy might apply differently to objects that infants have already had some exposure to, but not learned anew. Another challenge for this assumption is the short livability of this effect. If this enhanced performance within imbalanced frequency pairs for 8- to 10-month-old infants traces back to success due to mutual exclusivity, it is puzzling why older infants between 11- and 14-month of age should stop relying on this strategy and show worse performance. By contrast, given previous literature, the mutual exclusivity strategy flourishes with age (Bion et al., 2013) and is exploited across development up to adulthood (Halberda, 2006).

In sum, both possible explanations—the frequency imbalance cue and the exclusion strategy—hold limitations when applying them to the reported results. Especially, as we did not systematically manipulate our stimulus pairs, it is impossible to distinguish both potential explanations. Future studies should use a more controlled design and aim to detangle both explanations.

### No Noun Comprehension in 11- to 14-Month-Old Infants?

**Figure 5:**
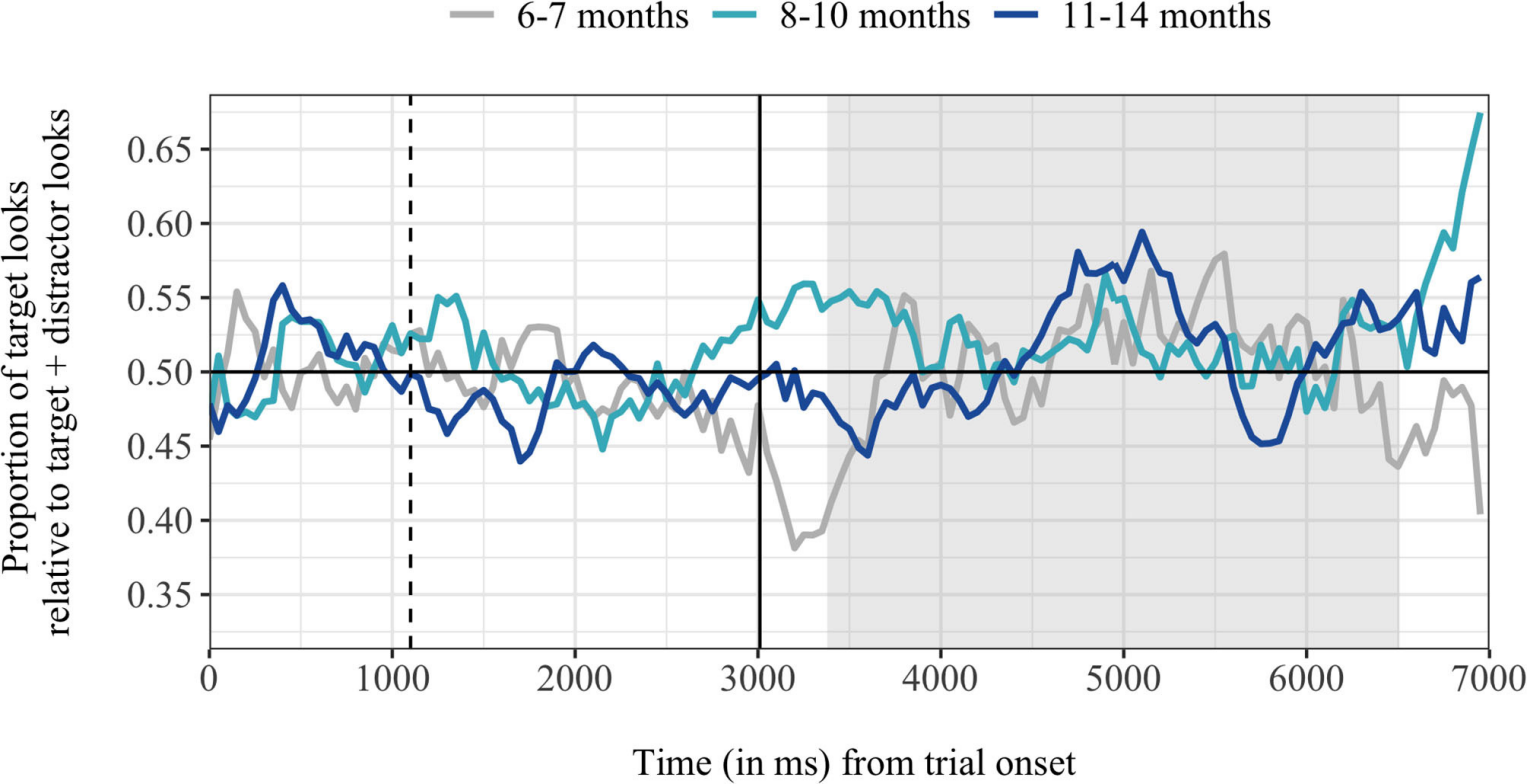
Infants mean proportion of looks to the target in relation to all looks to the screen (ordinate), averaged over the trial time course (abscissa). The vertical black lines indicate the onset of the carrier phrase (dashed) and target word (solid). Gray background indicates the time window of interest for all analyses. Horizontal black line indicates more looks to the target (above 0.50) or more looks to the distractor (below 0.50) in relation to all looks to the screen.

Surprisingly, even the oldest age group of 11- to 14 months-old infants failed to associate the common nouns with their referent. This is striking as the parental report indicated those infants understood 48% of our tested target words. Additionally, performance within this age group has been quite stable in previous LWL-studies (Bergelson and Swingley, 2012, 2015, 2018), and infants in this age span are assumed to show a non-linear improvement within their LWL-task performance (Bergelson, 2020). Indeed, when investigating the time course of the German-learning infants' target fixations (see age group 11–14 months in Figure 5), they did fixate the target picture more shortly with a peak around 2 s after hearing the target word. This peak overlaps with the previously suggested ideal time window to reveal target recognition (1,200–2,200 ms after target onset; Swingley, 2012), and was also identified within our cluster permutation analyses. The lack of evidence for word comprehension within the proportion index could be explained in terms of an inappropriately applied time window of interest. Older infants tend to get bored easily and might therefore fixate the distractor more after they have correctly identified the target picture (Fernald et al., 2008). From that perspective, the chosen time window of interest might have been too long and should be individually adapted for the wide range of age groups. Yet, determining the underlying window of interest is a tricky procedure, as different approaches modulate dependent variables, such as the proportion index (Fernald et al., 2008). Importantly, even though we find support for the above chance target looking within this age group within our cluster analysis, chances of observing the same effect by chance were high. This further underpins our conclusion that even the oldest infants between 11- and 14-months of age did not systematically fixate the targets more upon hearing them in the present study.

## Conclusion

Results of this LWL-study indicate that German-learning infants between 6- and 14-months failed to associate the tested nouns with their referents. Overall, especially for infants between 8- and 10-months of age, performance seems to be modulated by the frequency imbalance of the stimulus pairs. For stimulus pairs that highly differ within their word frequency, infants' performance was enhanced. This further emphasizes the already claimed assumption that frequency imbalance might serve as an additional extra-linguistic cue within the LWL-paradigm (Kartushina and Mayor, 2019) and extends respective evidence to a new target language, namely German. However, more work is needed to investigate the potential implications of the operationalization of word frequency and address other potential explanations, such as the exclusion strategy. Without doubt, further studies are needed to confirm or question the failure of German-learning infants to show robust noun-referent associations. Even though prior work supports a cross-cultural difference in the onset of word comprehension (Kartushina and Mayor, 2019; Frank et al., 2021), we think there is accumulating evidence that target language alone *should not be* considered as the sole explanation for this null effect. First and foremost, all alternative differences between those cross-cultural LWL-studies, such as the choice of stimulus material, should be explored. Importantly, by contributing data of a new target language and emphasizing potential influences of experimental parameters, as well as raising concerns about fundamental operationalization questions, the present work contributes to this ongoing process of elaborating and enhancing our understanding of the LWL-paradigm.

## Data Availability Statement

The datasets presented in this study can be found in online repositories. The names of the repository/repositories and accession number(s) can be found at: https://osf.io/72tjz/?view_only=bb78b0c0a3e74b4b94c4dbd92c971317.

## Ethics Statement

The studies involving human participants were reviewed and approved by Ethics Committee for Psychological Research, University of Tuebingen, Germany. Written informed consent to participate in this study was provided by the participants' legal guardian/next of kin.

## Author Contributions

CF and US conceived and designed the study. US organized the database. US and JS performed the statistical analysis. JS wrote the first draft of the manuscript. All authors contributed to manuscript revision, read, and approved the submitted version.

## Funding

This present work was supported by the Deutsche Forschungsgemeinschaft (DFG, German Research Foundation), within project FR 2519/7-1 of the research unit Modal and amodal cognition: Function and interaction (FOR 2718).

## Conflict of Interest

The authors declare that the research was conducted in the absence of any commercial or financial relationships that could be construed as a potential conflict of interest.

## Publisher's Note

All claims expressed in this article are solely those of the authors and do not necessarily represent those of their affiliated organizations, or those of the publisher, the editors and the reviewers. Any product that may be evaluated in this article, or claim that may be made by its manufacturer, is not guaranteed or endorsed by the publisher.

## Footnotes

^1^Note, that we tested additional 20 infants between 4 and 5 months of age. Data quality within the 18 monolingual infants was low. On average they provided only data to 2.88 of our 14 stimulus pairs, and 44% of infants provided not enough data to compute any proportion indices. Therefore, and for higher comparability to previous studies, we excluded the whole age group below 6 months from all further analysis.

^2^Parents of the participating infants were more highly educated than would be expected for a representative German sample. This reflects the selection bias in psychological research in general (Henrich et al., 2010) and in research on psychological development in particular (Nielsen et al., 2017).
