# Supplementary material for: No Evidence of Robust Noun-Referent Associations in German-Learning 6- to 14-Month-Olds
Source: Front Psychol. 2021 Oct 6;12:718742. doi: 10.3389/fpsyg.2021.718742 (PMC8526865; doi:10.3389/fpsyg.2021.718742)
Supplement: Supplementary file 1 [file Table_1.pdf]

Supplementary Material: *Appendix A*

Table A1

*Pictures presented as stimulus pairs are displayed next to each other. Target words (English Translation), average word frequency (6-7 months/8-10 months/11-14 months/**all**) and frequency imbalance of stimulus pairs (Δ) - based on parental vocabulary questionnaire and CHILDES database - are presented with each stimulus pair.*

Stimulus Pair	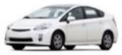	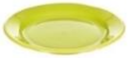	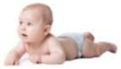	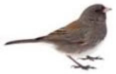	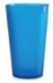	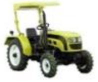
Target word	Auto (car)	Teller (plate)	Baby (baby)	Vogel (bird)	Becher* (cup)	Traktor (tractor)
Parental freq.	3.89/4.00/4.06/**4.00**	3.33/3.40/3.47/**3.41**	4.50/3.93/3.29/**3.80**	2.60/3.29/3.65/**3.27**	3.60/3.93/3.06/**3.50**	1.40/1.43/1.94/**1.63**
Parental imb	Δ = 0.59		Δ = 0.53		Δ = 1.88	
CHILDES freq	5771.00	493.00	1869.00	393.00	386.00	178.00
CHILDES imb	Δ = 5278.00		Δ = 1476.00		Δ = 208.00	

Stimulus Pair	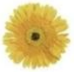	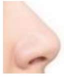	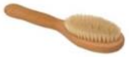	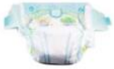	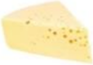	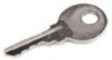
Target word	Blume (flower)	Nase (nose)	Bürste (brush)	Windel* (diaper)	Käse (cheese)	Schlüssel (key)
Parental freq.	2.40/2.50/3.35/**2.83**	3.60/4.07/4.27/**4.02**	1.60/2.27/2.65/**2.26**	4.90/4.71/4.53/**4.68**	2.30/2.33/2.62/**2.44**	3.00/3.85/3.47/**3.48**
Parental imb	Δ = 1.20		Δ = 2.42		Δ = 1.04	
CHILDES freq	285.00	955.00	39.00	394.00	334.00	260.00
CHILDES imb	Δ = 670.00		Δ = 355.00		Δ = 74.00	

Stimulus Pair	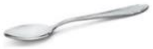	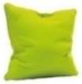	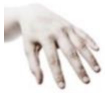	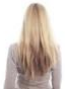	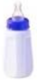	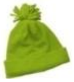
Target word	Löffel (spoon)	Kissen* (pillow)	Finger (finger)	Haare (hair)	Flasche (bottle)	Mütze (hat)
Parental freq.	4.10/4.29/4.35/**4.27**	2.56/2.13/2.69/**2.45**	3.90/3.64/3.29/**3.56**	3.80/3.92/4.06/**3.95**	4.00/3.79/3.35/**3.66**	3.60/3.86/3.65/**3.72**
Parental imb	Δ = 1.82		Δ = 0.39		Δ = 0.05	
CHILDES freq	659.00	91.00	611.00	426.00	743.00	292.00
CHILDES imb	Δ = 568.00		Δ = 185.00		Δ = 451.00	

Note. Words, that did *not* derive from the ELFRA-1 (Grimm and Doil, 2006) questionnaire are marked with an asterisk (*). Parental freq. ranged between 1 to 5 on a Likert-Scale, whilst CHILDES freq. reflects the absolute word count of the described sample out of the German CHILDES database (MacWhinney, 2000). Due to rounding errors within the parental freq. calculating over age group means does not exactly lead to mean frequency across the whole sample.

Table A1 (Continued)

*Pictures presented as stimulus pairs are displayed next to each other. Target words (English Translation), average word frequency (6-7 months/8-10 months/11-14 months/**all**) and frequency imbalance of stimulus pairs (Δ) - based on parental vocabulary questionnaire and CHILDES database - are presented with each stimulus pair*

Stimulus Pair	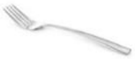	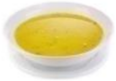	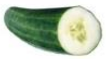	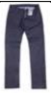	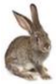	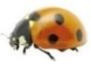
Target word	Gabel (fork)	Suppe (soup)	Gurke* (cucumber)	Hose (pants)	Hase (rabbit)	Käfer* (bug)
Parental freq.	2.50/2.80/3.24/**2.90**	1.10/1.43/1.12/**1.23**	3.22/3.13/2.94/**3.08**	4.00/3.60/4.24/**3.95**	2.20/2.87/2.59/**2.60**	1.90/2.20/1.53/**1.86**
Parental imb	Δ = 1.68		Δ = 0.88		Δ = 0.74	
CHILDES freq	140.00	158.00	39.00	988.00	698.00	350.00
CHILDES imb	Δ = 18.00		Δ = 949.00		Δ = 348.00	

Stimulus Pair	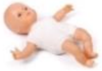	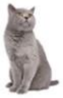	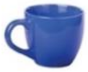	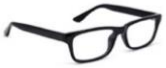
Target word	Puppe (doll)	Katze (cat)	Tasse (mug)	Brille (glasses)
Parental freq.	2.20/2.38/2.53/**2.40**	2.00/3.07/3.53/**3.00**	3.22/3.13/2.80/**3.08**	3.50/3.57/3.29/**3.44**
Parental imb	Δ = 0.60		Δ = 0.36	
CHILDES freq	1780.00	1662.00	430.00	311.00
CHILDES imb	Δ = 118.00		Δ = 119.00	

Note. Words, that did *not* derive from the ELFRA-1 (Grimm and Doil, 2006) questionnaire are marked with an asterisk (*). Parental freq. ranged between 1 to 5 on a Likert-Scale, whilst CHILDES freq. reflects the absolute word count of the described sample out of the German CHILDES database (MacWhinney, 2000). Due to rounding errors within the parental freq. calculating over age group means does not exactly lead to mean frequency across the whole sample.

References

Grimm, H., and Doil, H. (2006). ELFRA. Elternfragebögen für die Früherkennung von Risikokindern. Göttingen: Hogrefe Verlag.

MacWhinney, B. (2000). *The CHILDES Project: Tools for analyzing talk*. Third Edition. Mahwah, NJ: Lawrence Erlbaum Associates.
