# Supplementary material for: No Evidence of Robust Noun-Referent Associations in German-Learning 6- to 14-Month-Olds
Source: Front Psychol. 2021 Oct 6;12:718742. doi: 10.3389/fpsyg.2021.718742 (PMC8526865; doi:10.3389/fpsyg.2021.718742)
Supplement: Supplementary file 2 [file Data_Sheet_1.pdf]

Supplementary Material

Appendix B

On average our target words were reported as medium frequent (*M* = 3.29, *SD* = 0.85), on the five-point Likert-scale. To assure that low frequent target words did not account for our null finding, we excluded on an individual level all stimulus pairs where the parent reported their infant does not understand one or both target words (e.g. Kartushina and Mayor, 2019). This led to a loss of on average 2.43 stimulus pairs per infant (*SD* = 1.77 stimulus pairs, range = 0-7 stimulus pairs). Further, from the 42 infants, now additional 12 children provided less than four proportion indices to compute the PI-by-infant and were excluded from this analysis. The remaining sub-sample of 30 infants, was slightly younger than the original sample (*M* = 9.12 months, *SD* = 70.99 days) and consisted of six infants between 6- to 7-months, ten infants between 8- to 10-months and fourteen infants between 11- to 14-months. Within this sample, data quality was lower: On average 6.43 stimulus pairs were provided to calculate the PI-by-infant (*SD* = 2.18), while on average 49 % of infants provided data to compute the PI-by-item (*SD* = 3.45 infants). **(i) PI-by-infant** was positive for 17 out of 30 children (*M* = 0.023, *SD* = 0.16), and did not differ significantly from chance, V = 275, *p* = .39, BF_{01} = 3.18. **(ii) PI-by-item** was positive for 9 out of 13 stimulus pairs (*M* = 0.041, *SD* = 0.093). Note, that we excluded the stimulus pair fork-soup from all analyses, as only two children provided data to compute this proportion index ($\text{PI-by-item}_{\text{fork-soup}}$ = 0.28). Performance did not significantly differ from chance, V = 68.00, *p* = .13, BF_{01} = 1.33. Additionally, within this sub-sample again frequency imbalance (estimated over these 30 parental reports) was positively correlated to PI-by-item, *r*(11) = .44, albeit not significant, *p* = .067. However, Bayes Factor still indicates moderate evidence for such a positive correlation, BF_{01} = 0.27.

In sum, the previously observed result pattern upheld, as we did not find evidence for robust noun-referent associations in this sub-sample of German-learning infants. However, results from the PI-by-item suggest descriptively a higher performance within this sub-sample of infants including only words they had more than rare exposure to. We want to highlight, that comparison to our original results should be made with caution, as the sample drastically changed. Note, that the twelve infants we excluded, in sum still contributed 27 proportion indices to our stimulus pairs. It is striking, that out of these 27 proportion indices for stimulus pairs where both target words were reported as *more than rarely frequent* 74 % were negative. However, due to the low data contribution on an individual level we excluded these proportion indices (infants) from our analysis. Thus, this exclusion might partially explain the descriptively higher performance within this sub-sample. This could suggest that the enhanced performance might not be directly linked to the amount of word exposure these infants had, but rather go back on the differences within the sample.
